# Diverse Hits in De Novo Molecule Design: Diversity-Based Comparison of Goal-Directed Generators

**DOI:** 10.1021/acs.jcim.4c00519

**Published:** 2024-07-19

**Authors:** Philipp Renz, Sohvi Luukkonen, Günter Klambauer

**Affiliations:** † Johannes Kepler University Linz, Altenbergerstraße 69, Linz, AT 4040, Austria; ‡ Johannes Kepler University Linz, ELLIS Unit Linz, LIT AI Lab, Institute for Machine Learning, Altenbergerstraße 69, Linz, AT 4040, Austria

## Abstract

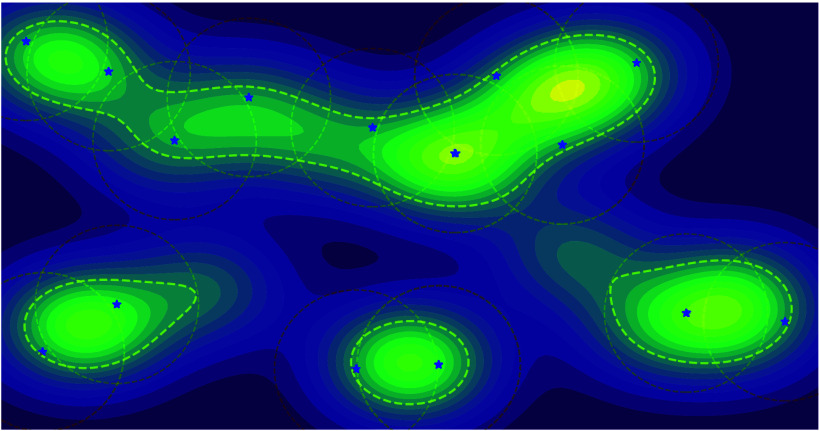

Since the rise of generative AI models, many goal-directed molecule generators have been proposed as tools for discovering novel drug candidates. However, molecule generators often produce highly similar molecules and tend to overemphasize conformity to an imperfect scoring function rather than capturing the true underlying properties sought. We rectify these two shortcomings by offering diversity-based evaluations using the #Circles metric and considering constraints on scoring function calls or computation time. Our findings highlight the superior performance of SMILES-based autoregressive models in generating diverse sets of desired molecules compared to graph-based models or genetic algorithms.

## Introduction

Goal-directed *de novo* drug design (DNDD) aims to generate small molecules possessing specific properties like efficacy, low toxicity, and drug-likeness,^1^ by exploring the vast space of drug-like molecules.^2^ This process involves generating novel chemical structures, guided by on-the-fly feedback from a scoring function to incorporate desired properties efficiently. With the surge of generative artificial intelligence, the field has witnessed a surge in interest, leading to the development of numerous new molecule generators, particularly those based on deep learning.^3−6^

Generating diverse sets of high-scoring molecules is essential in drug discovery. While most methods focus on producing individual high-scoring molecules, the reliance on quantitative structure–property relationship (QSPR) models introduces uncertainties and biases due to limited training data.^7^ These errors propagate to molecule generators, emphasizing the need for diverse molecule sets to enhance the chances of identifying successful drug candidates.^8−12^ Furthermore, diversity in molecule generation can lead to the exploration of novel chemical spaces beyond patented compounds.^13^ However, many existing generators suffer from ”mode collapse,” producing only a limited range of similar molecules.^12,14−17^ Various approaches have been proposed to address this issue and improve diversity in generated molecules.^16,18−23^

Previous comparative studies of molecule generators have often used insufficient diversity metrics. Well-known DNDD benchmarking platforms and leaderboards, such as GuacaMol^17^ and MOSES,^14^ include some classic diversity metrics: uniqueness and/or internal diversity, in the case of nongoal-directed molecule generation. But to our knowledge, there has been no systematic benchmark study of the capacity of different goal-directed molecule generators to generate a diverse set of high-scoring molecules.

**Figure 1:**
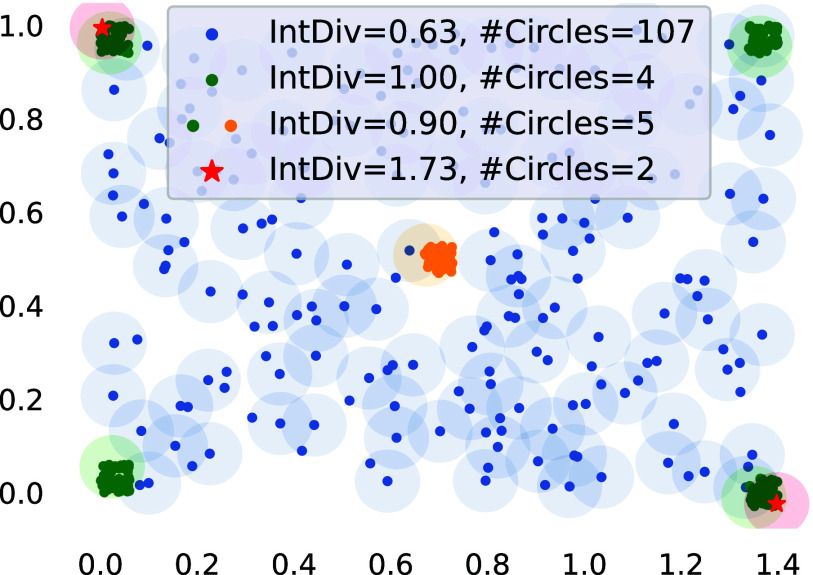
Comparison of internal diversity and #Circles. Internal diversity fails to capture high coverage of chemical space (blue), can be large for a few clusters of very similar molecules (green), and can decrease when adding additional molecules (green/yellow). IntDiv is maximized by two molecules with maximal distance (red). #Circles accurately captures the coverage of all sets.

Moreover, traditional metrics exhibit significant limitations in accurately characterizing the chemical space represented by a set of molecules.^24,25^ For example, in Figure 1 we show how the arguably most commonly used diversity metric, *internal diversity*, fails to capture coverage of chemical space. More simple metrics, like the fraction of unique molecules and unique Bemis-Murcko scaffolds^26^ are also inadequate as they can be optimized by generating many highly similar molecules, differing only in minor features. Recently diversity metrics based on sphere exclusion,^27^ such as *SEDiv*(28) and *#Circles*,^25^ have been proposed to quantify chemical space coverage. These metrics have been shown to align well with chemical intuition regarding the chemical diversity of known libraries and correlate well with the coverage of biological functionalities.

In addition to the aforementioned mode collapse problem, molecule generators tend to overoptimize molecules to their accessed scoring functions rather than to the actual desired properties.^7^ A high number of scoring function calls can lead to this overfitting to the biased QSPR models and a decline in molecule quality over the optimization cycles. Therefore, it is essential to evaluate and compare generators within a standardized computational budget, by restricting a) the overall compute time or b) the number of scoring function evaluations. Additionally, there’s growing interest in replacing machine learning-based scoring functions with more accurate but computationally expensive physics-based methods like docking.^28−31^ Generative methods that can efficiently learn from a few scoring function evaluations are preferable for such costly scoring functions. Gao et al.^32^ tested the sample efficiency of a range of generative methods given a constraint on the number of scoring function calls. Still, no studies focus on the generated molecules’ diversity under computational constraints.

In this work, we address the two shortcomings of previous comparisons, a) the insufficient diversity metrics and b) the generation without limitations on the computational budget. We systematically benchmark the performance of established molecule generators at generating diverse high-scoring molecules, referred to as *diverse hits*. We evaluate these generators within the framework of goal-directed optimization, where they operate under constraints such as a limited number of scoring function calls or time, emphasizing computational cost. We utilize the *#Circles* diversity metric as a key performance indicator, providing a comprehensive assessment of generative model efficiency in practical scenarios.

## Benchmark Setup

### Diverse Hits

We evaluate the performance of the tested generators based on the diversity of the generated high-scoring molecules. We define high-scoring molecules as ones with a score above a threshold *S* and refer to them as *hits*. We use the #Circles^25^ metric to measure diversity of the found hits. This metric counts the number of generated hits that are pairwise distinct by a distance threshold *D*. We refer to this as the number of *diverse hits*.

More specifically, given the set of generated hits, , the number of diverse hits is given by
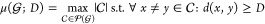
where  denotes the power set and *d*(*x*, *y*) is the distance between molecules *x* and *y*. This metric ensures that each found hit that is sufficiently different from those already found adds to the performance, and that hits similar to each other are not double-counted.

Figure 1 illustrates the computation of this metric as finding the largest set of circles centered on the molecules, such that no center lies within another circle. We provide a more detailed description of this metric in Supporting Information Section S1.

### Scoring Functions

#### Bioactivity Prediction Models

We evaluate the methods on three well-established molecule binary bioactivity label optimization tasks: JNK3,^33^ GSK3β,^33^ and DRD2.^16^ For each target we train a Random Forest classifier^34^ as a basis for our scoring functions. Table S1 provides details on the data sets and the performance of the predictive models. All scoring functions exhibit robust predictive performance, as indicated by their ROCAUC and Average Precision (AP) values.

During optimization, we use the classifier’s probabilistic activity output, *p*_RF_(*s*), as a scoring function. When predicting if a molecule is a hit, we adopt a score threshold of *S* = 0.5. Further details on the QSAR models are given in Supporting Information Section S2.1.

#### Property Filters

Generative models often generate molecules with high molecular weights (MW) or water-octanol partition coefficients (logP) and may contain idiosyncratic substructures, rendering them impractical for drug discovery projects,^7,35^ and often these molecules would be discarded in real-world applications. We address this issue by incorporating lenient property constraints into the scoring functions^35^ by defining acceptable ranges for MW ([157,761]Da) and logP ([-2.0,8.3]) values, and the fraction of idiosyncratic substructures ([0.00,0.08]). Further details on these filters are given in Supporting Information Section S2.2. During scoring, molecules violating any of these ranges have their score set to zero.

#### Diversity Filter

Most goal-directed molecule generators are not suitable for diverse generation out of the box, as they tend to get stuck in local optima of the scoring function.^12,14−17^ To enable diverse molecule generation, we enhance the scoring functions with the *diversity filter* (DF) from Blaschke et al.^16^ It assigns zero scores to molecules that are within a distance threshold *D*_DF_ = 0.7 to previously found hits. This approach prevents the optimization process from getting trapped in local optima and promotes the exploration of new chemical space regions. The DF proved to be crucial for performance in preliminary experiments and its use allows for the meaningful inclusion of generative algorithms originally designed for single-molecule optimization. A detailed description of the DF is given in Supporting Information Section S2.3.

The final scoring function is given by the product of the bioactivity model prediction, and the binary property and diversity filters.

### Compute Constraints

We evaluate the performance of the generators to create diverse hits under two computational constraint settings: (a) **Sample limit**, we limit the number of scoring function evaluations to 10K as proposed by Gao et al.,^32^ and (b) **Time limit**, we limit the time available to the algorithms to 600 s. All algorithms are executed using 8 cores of an AMD Ryzen Threadripper 1920X and a single NVidia RTX 2080 GPU.

### Generative Models

We utilize our benchmark setup to assess the following 12 methods. The methods were chosen based on their performance in previous benchmarks^17,32^ and to ensure that a range of methodically different approaches is included.

We test six LSTM-based autoregressive models operating on SMILES: **LSTM-HC**(36) optimizing with a hill-climb algorithm, **LSTM-PPO**(37) optimizing with the PPO algorithm, **Reinvent**(38) optimizing with the REINFORCE algorithm,^39^ and three extensions of Reinvent: **AugmentedHC** (mixture of Reinvent and hill-climb),^40^**AugMemory**,^41^ and BestAgentReminder (**BAR**).^42^ We also test three genetic algorithms making use of mutations of different molecular representations: **GraphGA**(43) operating on molecular graphs, **SmilesGA**(44) operating on SMILES, and **Stoned**(45) operating on SELFIES.^46^ We further test three models that generate molecules via sequential graph edits: **Mars**,^47^**Mimosa**,^48^ and **GFlowNet**/**GFlowNetDF**,^21^ which is tested with and without the DF as it supports diverse generation by default.

We compare these methods against two virtual screening (VS) baselines using the GuacaMol data set^17^ as a screening library. VS methods are deemed inefficient as they ignore feedback from already scored molecules but serve as a valuable baseline. The **VS Random** baseline evaluates the molecules with the scoring function in random order from the library. In contrast, the **VS MaxMin** baseline first sorts the molecules in the library with the MaxMin algorithm.^49^ This promotes diversity by ensuring that molecules screened first have as large pairwise distances as possible and prevents evaluating redundant molecules. Further details about these methods and the choice to exclude others are discussed in Supporting Information Section S3.

### Optimization

We conducted a hyperparameter search to optimize each combination of generative algorithm, scoring function, and computational constraint. Employing a random search with 15 trials per combination, we explored various hyperparameter ranges, and to assess result stability, we executed five independent runs with distinct random seeds. The selected hyperparameters are detailed in Supporting Information Table S2.

Throughout the optimizations, we tracked all generated molecules, their corresponding scores, and the generation time. This comprehensive recording prevents valuable molecules from being discarded unnecessarily. This is particularly crucial when using a diversity filter that steers the search away from already discovered solutions.

## Results and Discussion

**Figure 2:**
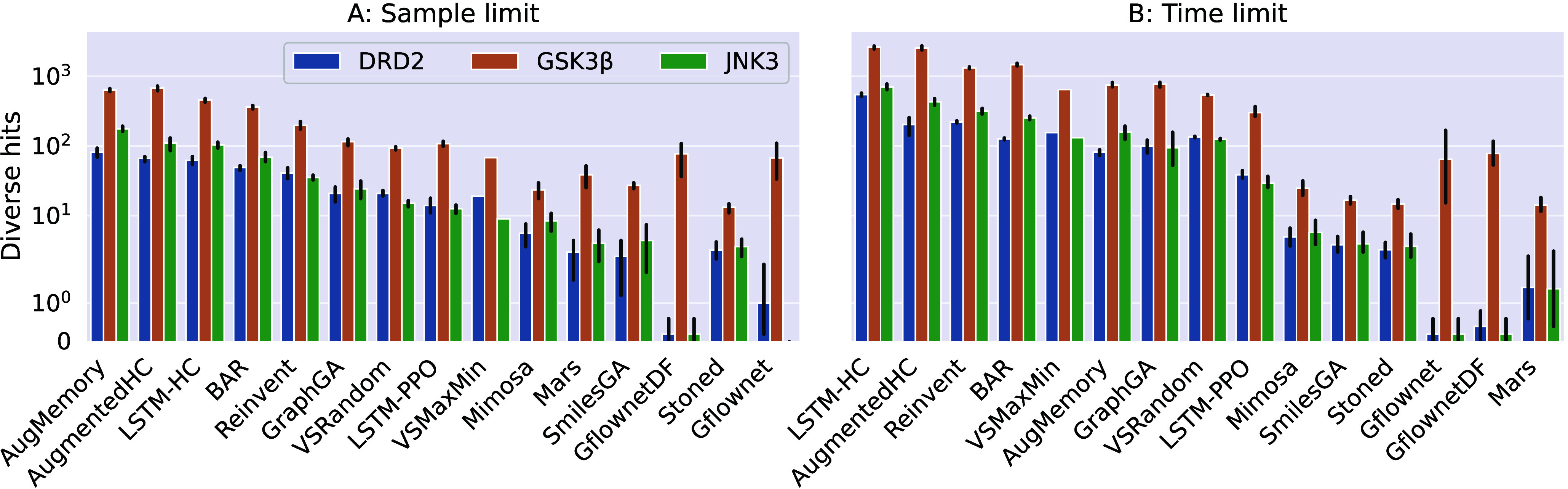
Number of diverse hits found by the tested methods (ordered by average rank) for the three studied optimization tasks. **A.** Results under a constraint of 10K scoring function evaluations. **B.** Results under a time constraint of 600 s. Error bars show the range of the results.

We benchmarked the capacity of a wide range of goal-directed molecule generators to design diverse hits under two computational constraint settings for three protein targets. The main results, i.e., the number of diverse hits under the constraints are shown in Figure 2 and discussed here. Extended results with complementary metrics are given in Supporting Information Section S5.

### Large Differences in Performance between Models and Tasks

Above all, we observe in Figure 2 a significant difference in the capacity to produce diverse hits between the different algorithms, tasks, and computational constraints. The number of diverse hits ranges from several molecules for Mars (worst) to several thousand molecules for LSTM-HC (best) in the time-constrained setting. We also see that the performance is highly task-dependent: all approaches find ∼10 × more diverse hits for GSK3β than DRD2/JNK3 with the most extreme difference between the GFlowNet generators. Finally, the absolute, as well as relative performance of the algorithms depends strongly on the used computational constraint and on the available compute budget as shown in Supporting Information Sections S5.2 and S5.3.

### SMILES-Based LSTM Models Perform Best in Generating Diverse Hits

Generally, the top ranks are dominated by autoregressive SMILES-based models. In the sample limit setting AugMemory performs best, making use of experience replay with selective purge and data augmentation, which leads to high sample efficiency. This allows the model to outperform its parent Reinvent. Similarly, the AugmentedHC model can also outperform its parent models Reinvent and LSTM-HC as shown in the original paper.^40^ LSTM-HC attains the third rank and can outperform Reinvent, which is in contrast to results in single molecule optimization tasks.^32^ The increase in performance of the extensions compared to their parent methods, comes with a significant computational cost as under the time limit the parent models are more competitive with their extensions, with LSTM-HC and Reinvent taking the top and third rank, respectively. We found that LSTM-HC is the most versatile algorithm achieving on average 84% of the top performance in each of the six settings (see Supporting Information Section S5.4). We think that this model class benefits from their stochastic generation policy which allows them to sample from different regions of chemical space at each optimization step. Thus, they can easily move to new regions once a new diverse hit has been found, resulting in high sample/compute efficiency.

### Limited Number of Diverse Hits with Graph-Based and Genetic Algorithms

The graph-based models generally occupy the lower ranks in this comparison. Among them, GraphGA is the only model that outperforms the virtual screening baselines. SmilesGA and Stoned both perform poorly in this comparison. Along with GraphGA, they are not able to match their competitive performance in the single molecule optimization tasks.^32^ We also found Mars and GFlowNet to perform poorly in this comparison, despite comparing well in previous diverse optimization studies.^21,25^ This discrepancy highlights the importance of a meaningful benchmark setup and comparison to models suited for diverse optimization. We hypothesize that genetic algorithms encounter challenges in diverse generation tasks as they traverse chemical space using incremental modifications to existing molecules. This might cause a slow transition to new high-scoring regions once a hit is found.

### Diversity at the Cost of Drug-likeness

**Figure 3:**
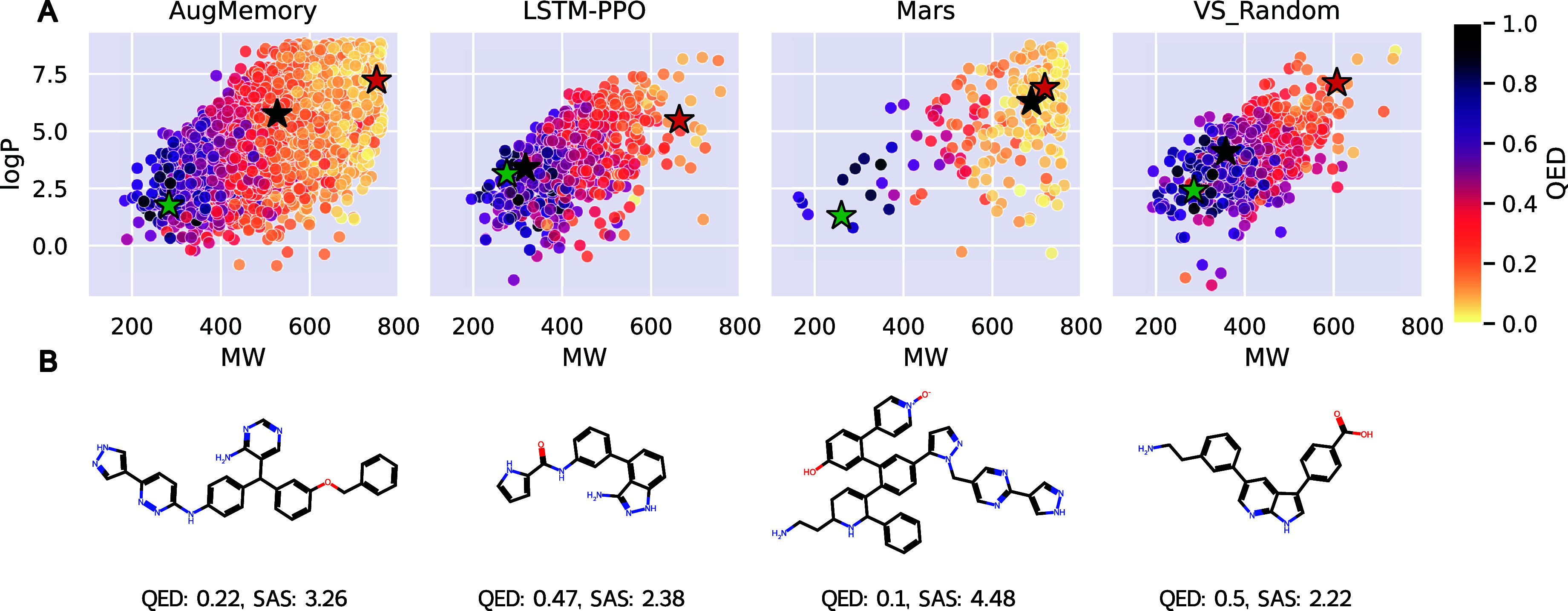
**A.** Chemical space cover by generated diverse hits by best (AugMemory), median (LSTM-PPO), and worst (Mars) methods, and the VS Random baseline, in the sample-constrained setting (all tasks combined). The green, black, and red stars represent the molecules with the highest, median, and lowest QED, respectively. **B.** Molecules with median QED.

We observed that some models achieve high diversity at the cost of drug-likeness. In Figure 3 we illustrate the chemical space covered and drug-likeness of the generated diverse hits by the best (AugMemory), median (LSTM-PPO), and worst (Mars) method, and the VS Random baseline, in the sample-constrained setting. We see that even though LSTM-PPO generates 10 times less diverse hits than AugMemory, these hits are generally more drug-like based on the QED score and overlap better with the VS baseline. In Supporting Information Section S5.5, we present distributions for additional properties of the generated diverse hits for all the methods. These distributions confirm that increased diversity is often achieved by generating larger, less drug-like molecules. We note that also the models achieving the lowest number of diverse hits (Mars and GFlowNet) struggle to generate drug-like molecules.

## Conclusion

In our study, we rigorously tested a range of molecule generators in diverse *de novo* design tasks, introducing a benchmark setup that overcomes limitations identified in previous studies. Our findings underscore the crucial importance of considering computational resources in the generation of molecules and employing a meaningful diversity measure in the context of DNDD.

We found that SMILES-based autoregressive models perform well, compared to graph-based models and genetic algorithms in generating diverse sets of high-scoring molecules, and that single molecule optimization performance does not necessarily translate to diverse optimization settings. Performance values range over several orders of magnitude for different models and tasks and compute constraints. The latter highlights the importance of considering the specific application and available resources when choosing a generative model for practical applications.

Due to the broad range of possible applications of generative models in drug discovery, this study cannot cover all relevant aspects in detail, such as the used scoring functions, synthesizability issues, or the amount of available compute budgets, which may drastically differ in real-world applications. Nevertheless, we believe that our findings will generalize to other settings, and that our benchmark setup will be useful for future studies in the field.

## Data Availability

The data, code, and instructions necessary to reproduce the results of this study are available for download at https://github.com/ml-jku/diverse-hits.

## Supporting Information Available

S1 - Diversity metric details, S2 - Scoring function details, S3 - Generative model details, S4 - Hyperparameter optimization, S5 - Extended results (PDF)
Additional drawings of generated structures (ZIP)
