# Supplementary material for: Diverse Hits in De Novo Molecule Design: Diversity-Based Comparison of Goal-Directed Generators
Source: J Chem Inf Model. 2024 Jul 19;64(15):5756–61. doi: 10.1021/acs.jcim.4c00519 (PMC11323242; doi:10.1021/acs.jcim.4c00519)
Supplement: Supplementary file 1 — ci4c00519_si_001.pdf [file ci4c00519_si_001.pdf]

Supporting Information
Diverse Hits in de novo Molecule Design: Diversity-based Comparison of Goal-directed Generators

Philipp Renz[1], Sohvi Luukkonen[2], and Günter Klambauer*[2]

[1]Johannes Kepler University Linz, Altenbergerstraße 69, Linz, AT 4040
[2]Johannes Kepler University Linz, ELLIS Unit Linz, LIT AI Lab, Institute for Machine Learning, Altenbergerstraße 69, Linz, AT 4040
*klambauer@ml.jku.at

S1 Diversity metric details

S1.1 Internal diversity

The internal diversity of a set of molecules is calculated as the average pairwise distance between all molecules in the set. For a set of molecules $\mathcal{M}$ it is calculated as,

$$\mathrm{IntDiv}(\mathcal{M}) = \frac{1}{|\mathcal{M}|(|\mathcal{M}|-1)} \sum_{m_i \in \mathcal{M}} \sum_{m_j \in \mathcal{M}} \mathrm{distance}(m_i, m_j). \quad (1)$$

As discussed in the main text, the internal diversity is a weak indicator of a set's diversity in the context of *de novo* molecule design. For instance, given a set of generated molecules, it can be maximized by selecting the two molecules with the highest pairwise distance. Additionally, internal diversity can be misleadingly high if the set consists of a few dense clusters of molecules that are far apart in molecular space. This scenario does not align with the goal of identifying many structurally diverse molecules.

When such a set is submitted to a biological assay, the information retrieved is highly redundant, making the assay costs unjustifiable relative to the information gained. Therefore we use the #Circles metric as the primary diversity metric in this study.

S1.2 #Circles metrics

The #Circles metric quantifies the diversity of a set of molecules. Given some distance metric in molecular space, such as the Tanimoto distance between fingerprint vectors, the #Circles metric is given by the maximal number of molecules with pairwise distances greater than a given threshold. This is equivalent to determining the packing number of the set of molecules in the distance metric space.

Alternatively, the #Circles metric can be interpreted as the size of a graph's Maximum Independent Set (MIS). In this context, given a set of molecules and a distance threshold, a graph is constructed where each molecule corresponds to a node, and edges connect nodes if the distance between the respective molecules is below the threshold. The MIS of this graph is the largest subset of nodes with no two nodes connected by an edge. Thus, the #Circles metric equals the size of the graph's MIS.

Calculating the packing number or MIS is generally NP-hard but can be approximated efficiently using a greedy algorithm. One simple, linear-time greedy algorithm is the sphere exclusion method. This algorithm iterates through the set of molecules, adding a molecule to the selected set if it is not within the distance threshold of any already selected molecule. This algorithm iterates through the set of molecules and adds a molecule to the set of selected molecules if it is not within the distance threshold of any of the already selected molecules.

The MaxMin algorithm offers a sub-quadratic time approximation for the packing number. Instead of iterating through the list in order, it selects the molecule that is furthest from the already selected molecules, specifically the one with the greatest minimum distance to the selected

set. This process can be further accelerated from quadratic to sub-quadratic time using an Alpha-beta pruning-like strategy.

S1.3 Diverse hits

To compute the number of diverse hits, we use the score threshold of $S = 0.5$, the Tanimoto distance between Morgan fingerprints (radius=2, size=2048) as the distance metric, and a distance threshold of $D = 0.7$, which aligns with the sharp drop in the probability of similar bioactivities beyond this value[1–3]. We efficiently approximate #Circles using the MaxMin algorithm[2].

S2 Scoring function details

S2.1 Bioactivity prediction models

For each of the datasets, we partition the data into training and test sets using a random 75/25% split and build a Random Forrest classifier[4] with the sklearn (v1.3.0) default parameters on Morgan fingerprints (radius=2, size=2048)[5].

In Table S1 we show the classification performance of the predictive models on the respective test sets. We evaluated the classification performance for the classifiers using the Area under the Receiver Operating Characteristic Curve (ROCAUC) and Average Precision (AP) metrics, which show good performance for all three datasets. We also report the Precision and Recall at the score threshold, $S = 0.5$, used in the molecule optimization tasks. Table S1 also shows the number of diverse hits in the training set giving a lower bound on the number of diverse hits recoverable by the generators.

We establish a score threshold of $S = 0.5$ for the optimization tasks. Precision values are high at this threshold, indicating that compounds scoring above this mark are very likely to be true actives. At the same time, the recall values are not excessively low, indicating that not too many true actives are rejected. Increasing the score threshold to a higher value like 0.9 would result in marginally higher precision values but drastically reduced recall, and would result in the discarding of many potentially active compounds. Furthermore using a high score threshold biases the optimization process towards recovering active compounds in the training set[6].

Table S1: Performance Metrics for JNK3, GSK3β and DRD2 activity prediction models. The table shows the ROCAUC, Average Precision (AP), Precision, and Recall at a 0.5 threshold, average scores of train and test actives, the number of samples, and the number of diverse hits in the training sets .

Target	ROCAUC	AP	Prec@0.5	Rec@0.5	Prec@0.9	Rec@0.9	#Samples	DivHits
JNK3	0.96	0.86	0.97	0.62	0.98	0.21	50390	220
GSK3	0.98	0.93	0.98	0.72	0.99	0.32	52802	643
DRD2	1.00	0.91	0.89	0.79	0.97	0.30	102981	229

S2.2 Property filters

Generative models have been observed to frequently produce compounds that feature atypical substructures or yield compounds with exceptionally high molecular weight (MW) or water-octanol partition coefficients (logP) values[6,7]. To enable a meaningful comparison we constrain the optimization objective to ensure that the generated molecules have properties within the range of those in a reference set. We use the ChEMBL subset provided in GuacaMol[8] as a reference set. In line with[7], we use relatively lenient property constraints to ensure that the optimization objective is not overly restrictive but ensures that compounds with strongly atypical properties are not rewarded. For the molecular weight (MW) and water-octanol partition coefficient (logP), we determine two-sided quantile-based lower and upper bounds, ensuring that 99% of the compounds in the ChEMBL dataset fall within these limits. For MW this results in a permissible range of $[157, 761]$ Da, and for logP this range is $[-2.0, 8.3]$.

We adopt the methodology outlined by[7] to address the challenge of uncommon substructures. We first partition the GuacaMol data set into a reference set and a calibration set, comprising 1.3M and 300K compounds, respectively. Then, we compute the unfolded ECFP4 fingerprints[5] for all compounds within the reference set, and determine the set of all occurring hash values. This

gives a reference of substructures typically found in drug-like molecules. We then compute the fingerprints for each compound in the calibration set and calculate the proportion of substructures absent in the reference set. We determine a threshold for this proportion such that 99% of the compounds in the calibration set fall below it, which evaluates to 0.08.

S2.3 Diversity filter

The diversity filter (DF) algorithm is given in Algorithm 1. The DF is initialized using an empty list M. Whenever a compound c is generated, it is compared with all compounds in M. If its distance to any compound in M is less than D_{DF} the compound does not pass the diversity filter and its DF-score is set to zero. If a compound passes the diversity filter, its DF-score is set to one. If a compound passes the diversity filter and $s(c) > s_{\mathrm{DF}}$ it is added to M.

For the experiments in this study, we set $D_{\mathrm{DF}} = 0.7$ and $s_{\mathrm{DF}} = 0.5$. We do not make explicit use of the bucket mechanism originally proposed[9]. Instead, we set the bucket size to one, as this resulted in quicker reorientation and faster exploration in preliminary experiments.

Algorithm 1: Computation of DF-score

Input : Generated compound c, score of compound $s(c)$, Filter list M, DF score threshold s_{DF}, DF distance threshold D_{DF}

Output: DF score s_{DF}, Updated filter list M

```
passes ← True ;                          // Initialize pass status to True
for each compound m in M do
    if distance(c, m) < D_DF then
        passes ← False ;                 // Set pass status to False
        break
if passes and s(c) > S then
    Add c to M ;                         // Add compound to filter list
return passes, M
```

S3 Generative model details

The tested models include a range of auto-regressive models operating on SMILES strings[10]. **LSTM-HC**[11] uses a hill-climb algorithm for fine-tuning a pre-trained LSTM model. **Reinvent**[12] optimizes a recurrent neural network using the REINFORCE algorithm[13] combined with prior regularization. **AugmentedHC**[14] forms a hybrid between the Reinvent and LSTM-HC models, by only using the Reinvent loss of the k top-scoring compounds to update the model. **AugMemory**[15] extends Reinvent, adding experience replay and data augmentation to increase sample efficiency. It makes use of selective memory purge to make experience replay compatible with the diversity filter described below. The BestAgentReminder (**BAR**) method[16] also extends the Reinvent algorithm, by keeping track of the best agent found so far and intersperses samples from the best agent with samples from the current model to stabilize training. **LSTM-PPO**[17] makes use of the popular PPO reinforcement learning algorithm to tune the model[18].

We test three genetic algorithms making use of mutations of different molecular representations. **GraphGA**[19] is a graph-based genetic algorithm that operates on the graph representation of molecules, and has shown competitive performance in previous benchmarks[8,20]. **SmilesGA**[21] generates novel molecules by encoding SMILES strings[10] into production rules of a context-free grammar and randomly inserting mutations into these rules. **Stoned**[22] generates molecules by introducing point mutations into the SELFIES[23] representation of molecules. All three genetic algorithms used randomly selected starting compounds from the Guacamol dataset[8].

We further test a range of models that generate molecules via sequential graph edits. **Mars**[24] makes use of Markov chain Monte Carlo sampling to generate molecules and achieved the best performance in a previous diverse optimization evaluation[25]. **Mimosa**[26] also operates on the graph representation of molecules and evolves molecules by applying a sequence of graph edits. **GFlowNet**[27] similarly sequentially builds molecules by graph edits but uses a specialized learning objective to enable diverse candidate generation.

While a range of strategies to promote diversity has been suggested[9,25,28–31], the diversity filter proposed in[9] is emerging as a standard approach employed in many studies[7,14,15,29] as it is a natural solution in line with our optimization objective, and is easy to combine with different generative models. Therefore we focus on the use of the DF for inducing diversity into the generated molecules. As GFlowNet is designed for diverse optimization we test it both with and without the diversity filter.

We do not test some other methods designed for diverse optimization, as they are conceptually similar to prior regularization used in Reinvent and its derivatives. We provide a detailed discussion in Section S3.2.

S3.1 Virtual screening baselines

We also test two virtual screening (VS) baselines that sample molecules the from the Guacamol dataset[8]. VS methods are deemed inefficient as they ignore feedback from already scored molecules, but serve as a valuable baseline. **VS Random** scores this library in random order.

We also test **VS MaxMin** which is adapted to diverse optimization. This algorithm operates on the screening library sorted by the MaxMin algorithm[2]. This algorithm first selects a random compound and then it selects the compound with the largest distance from the first one. The algorithm next selects the compound that is maximally distant from already selected ones. It does so by selecting the compound with the maximal minimum distance to already selected ones. This promotes diversity by ensuring that molecules screened first have as large pairwise distances as possible and prevents evaluating redundant molecules.

S3.2 Choice of tested algorithms

We did not test the following algorithms that have been reported to help improve the diversity of generated molecules. We did not include double-loop reinforcement learning[29] as it is conceptually very similar to the Augmented Memory algorithm[15]. Both algorithms use augmented versions of previously generated compounds to update the generative model multiple times.

We did not test the exploration approaches taken in[28,30] as they are conceptually similar to the prior regularization approaches that are used in Reinvent[12] and its descendants Augmented Hill-Climb[14] and Augmented Memory[15]. We also did not include the the method proposed in[32] in our experiments, as it is similar to the genetic algorithm equipped with the diversity filter.

Active learning methods have been shown to be effective in increasing the sample efficiency in optimization tasks[33,34]. Testing these methods is beyond the scope of this study, as implementation

and tuning is non-trivial. However, learning a fast proxy scoring function effectively reduces the cost of scoring molecules. Therefore the time constraint experiments give an indication of the performance of active learning methods. In principle, all the methods tested in this study can be combined with active learning methods, and pose an interesting avenue for future research.

S4 Hyperparameter optimization

For each generative model, we performed hyperparameter optimization to identify the best-performing hyperparameters for each combination of a generative algorithm, scoring function, and the used compute constraint. For each combination, we performed 15 runs with independently sampled hyperparameters. The hyperparameter distributions used for the random search and the selected parameters are given in Table S2.

In principle, the performance of the Reinvent derivatives AugmentedHC, AugmentedMem, and BAR could match that of Reinvent, when selecting the right hyperparameters. However, in this comparison, we restricted the parameter ranges to ensure non-trivial differences between the algorithms. This allows us to analyze the impact of the modifications in this setting.

Table S2: Hyperparameter ranges for the tested optimizers. We executed a random search using the distributions specified in this table. The selected hyperparameters for the respective constraint settings and targets are shown in the last six columns.

		Limit	Samples			Time		
Optimizer	Parameter	Search Space	DRD2	GSK3β	JNK3	DRD2	GSK3β	JNK3
AugHC	`batch_size`	RandInt(128, 512)	482	305	510	440	487	321
	`learning_rate`	LogUniform($10^{-4}, 10^{-3}$)	3.55e−4	2.89e−4	3.39e−4	2.24e−4	2.65e−4	1.90e−4
	`sigma`	Uniform(100.0, 500.0)	432.90	412.21	468.84	201.75	358.36	183.90
	`topk`	Uniform(0.15, 0.35)	0.16	0.17	0.17	0.20	0.24	0.17
AugMemory	`augmentation_rounds`	RandInt(1, 7)	6	6	2	4	1	1
	`batch_size`	RandInt(32, 128)	110	126	41	114	125	91
	`learning_rate`	LogUniform($10^{-4}, 10^{-3}$)	1.56e−4	1.25e−4	2.00e−4	1.61e−4	7.55e−4	3.90e−4
	`replay_buffer_size`	RandInt(32, 128)	107	82	111	111	74	61
	`sigma`	Uniform(100.0, 500.0)	409.80	369.46	332.04	493.91	490.70	261.11
BestAgentReminder	`alpha`	Uniform(0.3, 0.7)	0.67	0.42	0.34	0.45	0.42	0.53
	`batch_size`	RandInt(16, 256)	95	221	177	111	221	179
	`learning_rate`	LogUniform($10^{-4}, 10^{-3}$)	2.07e−4	2.58e−4	8.64e−4	3.12e−4	2.58e−4	1.25e−4
	`sigma`	Uniform(100.0, 500.0)	430.72	370.18	476.31	160.91	370.18	493.97
GA	`mutation_rate`	LogUniform($10^{-3}, 10^{-1}$)	3.96e−3	2.13e−3	1.86e−2	1.97e−2	4.91e−3	1.86e−2
	`offspring_size`	RandInt(50, 500)	160	426	230	245	130	230
	`population_size`	RandInt(50, 500)	217	397	409	444	496	409
Gflownet	`learning_rate`	LogUniform($10^{-5}, 10^{-3}$)	5.98e−5	1.72e−4	3.52e−4	1.17e−5	1.72e−4	3.33e−5
	`momentum`	Uniform(0.5, 0.9)	0.72	0.72	0.68	0.82	0.72	0.70
	`sampling_tau`	Uniform(0.8, 0.99)	0.87	0.96	0.83	0.97	0.96	0.93
GflownetDF	`learning_rate`	LogUniform($10^{-5}, 10^{-3}$)	8.25e−4	9.30e−4	1.37e−4	8.25e−4	3.75e−5	2.71e−5
	`momentum`	Uniform(0.5, 0.9)	0.83	0.78	0.60	0.83	0.77	0.57
	`sampling_tau`	Uniform(0.8, 0.99)	0.98	0.83	0.91	0.98	0.81	0.98
LSTM-HC	`mols_to_sample`	RandInt(8, 2048)	174	245	463	1695	859	1503
	`optimize_n_epochs`	RandInt(1, 6)	4	1	1	1	1	1
LSTM-PPO	`batch_size`	RandInt(64, 1024)	508	179	401	948	508	401
	`clip_param`	Uniform(0.1, 0.6)	0.11	0.19	0.41	0.12	0.22	0.41
	`entropy_weight`	Uniform(0.01, 1.0)	0.49	0.48	0.25	0.16	0.14	0.25
	`episode_size`	RandInt(64, 4096)	2277	1913	1121	2661	1932	1121
	`kl_div_weight`	RandInt(1, 10)	5	3	6	2	3	6
Mars	`batch_size`	RandInt(64, 512)	434	418	129	434	86	129
	`n_layers`	RandInt(1, 4)	1	3	2	1	1	2
	`num_mols`	RandInt(32, 512)	248	83	371	248	239	371
Mimosa	`lamb`	Uniform(0.1, 10.0)	7.07	5.84	2.03	7.07	5.84	1.73
	`population_size`	RandInt(50, 200)	150	199	87	150	199	85
	`train_epoch`	RandInt(1, 10)	2	2	9	2	2	8
Reinvent	`batch_size`	RandInt(256, 512)	260	288	417	462	454	464
	`experience_replay`	RandInt(0, 64)	49	34	54	38	12	50
	`learning_rate`	LogUniform($10^{-5}, 10^{-2}$)	1.00e−3	1.56e−3	3.25e−4	2.65e−4	2.24e−3	2.15e−4
	`sigma`	Uniform(100.0, 600.0)	582.10	540.86	296.22	357.38	244.20	283.52
SmilesGA	`gene_size`	RandInt(100, 600)	370	590	361	370	374	361
	`n_mutations`	RandInt(100, 300)	214	165	176	214	253	176
	`population_size`	RandInt(50, 200)	101	169	111	101	89	111
Stoned	`generation_size`	RandInt(50, 1000)	461	872	980	461	872	980

Table S3: Performance for the tested methods under a sample limit. Performance is given by the number of hits, diverse hits (DivHits), novel diverse hits (NDivHits), and internal diversity (IntDiv). The internal diversity is calculated on the discovered hits.

	DRD2			GSK3β			JNK3		
	DivHits	NDivHits	IntDiv	DivHits	NDivHits	IntDiv	DivHits	NDivHits	IntDiv
AugMemory	$81^{\pm19\%}$	$9^{\pm75\%}$	$0.76^{\pm0.01}$	$636^{\pm6\%}$	$507^{\pm12\%}$	$0.82^{\pm0.00}$	$176^{\pm11\%}$	$104^{\pm13\%}$	$0.77^{\pm0.00}$
AugmentedHC	$66^{\pm11\%}$	$3^{\pm44\%}$	$0.77^{\pm0.01}$	$674^{\pm11\%}$	$533^{\pm11\%}$	$0.84^{\pm0.00}$	$111^{\pm27\%}$	$63^{\pm41\%}$	$0.79^{\pm0.01}$
LSTM-HC	$62^{\pm16\%}$	$8^{\pm37\%}$	$0.76^{\pm0.01}$	$456^{\pm9\%}$	$231^{\pm16\%}$	$0.84^{\pm0.01}$	$103^{\pm13\%}$	$36^{\pm17\%}$	$0.78^{\pm0.00}$
BAR	$49^{\pm11\%}$	$1^{\pm56\%}$	$0.77^{\pm0.02}$	$361^{\pm8\%}$	$156^{\pm11\%}$	$0.85^{\pm0.00}$	$69^{\pm20\%}$	$20^{\pm19\%}$	$0.79^{\pm0.00}$
Reinvent	$41^{\pm25\%}$	$3^{\pm53\%}$	$0.74^{\pm0.02}$	$198^{\pm18\%}$	$135^{\pm24\%}$	$0.81^{\pm0.01}$	$35^{\pm11\%}$	$6^{\pm68\%}$	$0.75^{\pm0.01}$
GraphGA	$21^{\pm31\%}$	$7^{\pm55\%}$	$0.75^{\pm0.04}$	$115^{\pm14\%}$	$78^{\pm15\%}$	$0.84^{\pm0.00}$	$24^{\pm37\%}$	$10^{\pm61\%}$	$0.79^{\pm0.01}$
VSRandom	$21^{\pm12\%}$	$0^{\pm0\%}$	$0.82^{\pm0.01}$	$93^{\pm6\%}$	$7^{\pm12\%}$	$0.87^{\pm0.00}$	$15^{\pm13\%}$	$0^{\pm0\%}$	$0.83^{\pm0.01}$
LSTM-PPO	$14^{\pm32\%}$	$0^{\pm0\%}$	$0.81^{\pm0.02}$	$108^{\pm9\%}$	$16^{\pm26\%}$	$0.87^{\pm0.00}$	$13^{\pm18\%}$	$1^{\pm71\%}$	$0.81^{\pm0.02}$
VSMaxMin	$19^{\pm0\%}$	$0^{\pm0\%}$	$0.88^{\pm0.00}$	$68^{\pm0\%}$	$8^{\pm0\%}$	$0.89^{\pm0.00}$	$9^{\pm0\%}$	$0^{\pm0\%}$	$0.88^{\pm0.00}$
Mimosa	$6^{\pm47\%}$	$0^{\pm224\%}$	$0.80^{\pm0.06}$	$23^{\pm33\%}$	$8^{\pm62\%}$	$0.84^{\pm0.02}$	$8^{\pm37\%}$	$3^{\pm49\%}$	$0.78^{\pm0.07}$
Mars	$3^{\pm62\%}$	$0^{\pm224\%}$	$0.39^{\pm0.26}$	$39^{\pm45\%}$	$36^{\pm49\%}$	$0.81^{\pm0.02}$	$4^{\pm64\%}$	$2^{\pm84\%}$	$0.61^{\pm0.09}$
SmilesGA	$3^{\pm80\%}$	$0^{\pm224\%}$	$0.64^{\pm0.36}$	$27^{\pm12\%}$	$14^{\pm26\%}$	$0.85^{\pm0.01}$	$4^{\pm83\%}$	$2^{\pm87\%}$	$0.58^{\pm0.34}$
GflownetDF	$0^{\pm224\%}$	$0^{\pm224\%}$	$0.00^{\pm0.00}$	$77^{\pm60\%}$	$73^{\pm61\%}$	$0.81^{\pm0.00}$	$0^{\pm224\%}$	$0^{\pm224\%}$	$0.00^{\pm0.00}$
Stoned	$3^{\pm41\%}$	$0^{\pm0\%}$	$0.62^{\pm0.15}$	$13^{\pm19\%}$	$1^{\pm64\%}$	$0.79^{\pm0.06}$	$4^{\pm37\%}$	$0^{\pm224\%}$	$0.56^{\pm0.15}$
Gflownet	$1^{\pm122\%}$	$0^{\pm0\%}$	$0.15^{\pm0.33}$	$67^{\pm75\%}$	$64^{\pm77\%}$	$0.81^{\pm0.01}$	$0^{\pm0\%}$	$0^{\pm0\%}$	$0.00^{\pm0.00}$

Table S4: Performance for the tested methods under a time limit. Performance is given by the number of hits, diverse hits (DivHits), novel diverse hits (NDivHits), and internal diversity (IntDiv). The internal diversity is calculated on the discovered hits.

	DRD2			GSK3β			JNK3		
	DivHits	NDivHits	IntDiv	DivHits	NDivHits	IntDiv	DivHits	NDivHits	IntDiv
LSTM-HC	$544^{\pm7\%}$	$154^{\pm15\%}$	$0.80^{\pm0.00}$	$2620^{\pm7\%}$	$2045^{\pm8\%}$	$0.84^{\pm0.00}$	$708^{\pm13\%}$	$487^{\pm13\%}$	$0.81^{\pm0.00}$
AugmentedHC	$214^{\pm31\%}$	$50^{\pm42\%}$	$0.80^{\pm0.01}$	$2543^{\pm9\%}$	$2251^{\pm10\%}$	$0.85^{\pm0.00}$	$433^{\pm14\%}$	$291^{\pm15\%}$	$0.81^{\pm0.00}$
Reinvent	$221^{\pm5\%}$	$48^{\pm16\%}$	$0.79^{\pm0.01}$	$1315^{\pm4\%}$	$1100^{\pm5\%}$	$0.84^{\pm0.00}$	$318^{\pm11\%}$	$184^{\pm17\%}$	$0.79^{\pm0.01}$
BAR	$126^{\pm4\%}$	$7^{\pm30\%}$	$0.80^{\pm0.01}$	$1469^{\pm6\%}$	$1022^{\pm10\%}$	$0.85^{\pm0.00}$	$252^{\pm7\%}$	$132^{\pm15\%}$	$0.80^{\pm0.01}$
VSMaxMin	$155^{\pm0\%}$	$0^{\pm0\%}$	$0.83^{\pm0.00}$	$643^{\pm0\%}$	$88^{\pm0\%}$	$0.87^{\pm0.00}$	$131^{\pm0\%}$	$3^{\pm0\%}$	$0.83^{\pm0.00}$
AugMemory	$82^{\pm11\%}$	$7^{\pm44\%}$	$0.75^{\pm0.01}$	$753^{\pm9\%}$	$615^{\pm12\%}$	$0.82^{\pm0.01}$	$163^{\pm26\%}$	$77^{\pm33\%}$	$0.78^{\pm0.01}$
GraphGA	$102^{\pm27\%}$	$50^{\pm21\%}$	$0.78^{\pm0.01}$	$774^{\pm10\%}$	$699^{\pm11\%}$	$0.85^{\pm0.00}$	$111^{\pm56\%}$	$70^{\pm68\%}$	$0.80^{\pm0.01}$
VSRandom	$134^{\pm3\%}$	$0^{\pm0\%}$	$0.82^{\pm0.00}$	$540^{\pm2\%}$	$86^{\pm4\%}$	$0.87^{\pm0.00}$	$125^{\pm4\%}$	$4^{\pm12\%}$	$0.83^{\pm0.00}$
LSTM-PPO	$39^{\pm18\%}$	$0^{\pm0\%}$	$0.81^{\pm0.01}$	$308^{\pm25\%}$	$69^{\pm48\%}$	$0.87^{\pm0.00}$	$30^{\pm28\%}$	$2^{\pm130\%}$	$0.82^{\pm0.00}$
Mimosa	$5^{\pm34\%}$	$0^{\pm0\%}$	$0.82^{\pm0.04}$	$26^{\pm33\%}$	$8^{\pm68\%}$	$0.84^{\pm0.02}$	$6^{\pm50\%}$	$1^{\pm71\%}$	$0.74^{\pm0.09}$
SmilesGA	$4^{\pm35\%}$	$0^{\pm224\%}$	$0.76^{\pm0.11}$	$17^{\pm17\%}$	$8^{\pm33\%}$	$0.84^{\pm0.04}$	$4^{\pm59\%}$	$2^{\pm82\%}$	$0.42^{\pm0.40}$
Stoned	$3^{\pm34\%}$	$0^{\pm0\%}$	$0.54^{\pm0.30}$	$15^{\pm18\%}$	$1^{\pm96\%}$	$0.76^{\pm0.06}$	$4^{\pm50\%}$	$0^{\pm224\%}$	$0.61^{\pm0.17}$
Gflownet	$0^{\pm224\%}$	$0^{\pm224\%}$	$0.00^{\pm0.00}$	$112^{\pm70\%}$	$108^{\pm73\%}$	$0.80^{\pm0.01}$	$0^{\pm224\%}$	$0^{\pm224\%}$	$0.00^{\pm0.00}$
GflownetDF	$0^{\pm137\%}$	$0^{\pm224\%}$	$0.00^{\pm0.00}$	$87^{\pm53\%}$	$84^{\pm51\%}$	$0.81^{\pm0.01}$	$0^{\pm224\%}$	$0^{\pm224\%}$	$0.00^{\pm0.00}$
Mars	$2^{\pm100\%}$	$0^{\pm0\%}$	$0.37^{\pm0.34}$	$15^{\pm36\%}$	$10^{\pm45\%}$	$0.75^{\pm0.03}$	$2^{\pm122\%}$	$0^{\pm224\%}$	$0.19^{\pm0.19}$

S5 Extended results

S5.1 Additional metrics

Tables S3 and S4 give extended results for both constraints settings, including the number of hits, novel diverse hits, and internal diversity of the found hits. The novel diverse hits are calculated as follows: First, we take the hits found by the generative model and from these remove all compounds that have a distance of less than 0.7 to any active compound in the training set. Then we calculate the #Circles metric on this reduced set. While the number of novel diverse hits is in general highly correlated with the number of diverse hits, the virtual screening methods generate relatively few novel diverse hits. This is because the virtual screening methods are biased toward finding compounds that are similar to the training set, which can be seen in similar distributions of the molecular properties of the generated molecules and the training set (see Figures S6 & S7).

Figure S1 shows the correlation between different diversity metrics, namely the number of hits, diverse hits, the number of novel diverse hits, and the internal diversity of the hits. We can see that the number of diverse hits is strongly correlated with the number of novel diverse hits. The internal diversity is only weakly correlated with these metrics. This means internal diversity values reported in previous studies are not a good indicator of the number of diverse (novel) hits found.

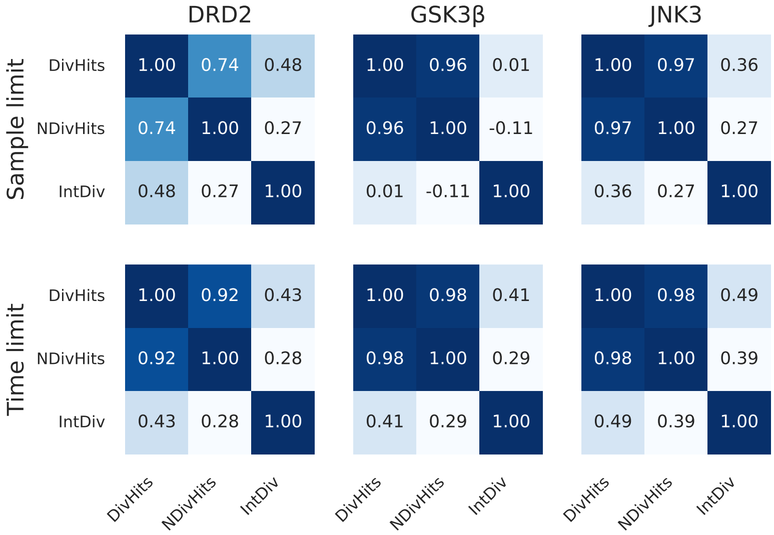

Figure S1: Correlation between different diversity metrics. The number of diverse hits is correlated with the number of novel diverse hits. The internal diversity is only weakly correlated with the other metrics.

S5.2 Optimization curves

Figures S2 and S3 show the number of diverse hits found by the tested methods over the number of scoring function evaluations and the time elapsed. Most methods show no sign of saturating performance within the chosen limits. This shows that the comparison of the methods is only meaningful under standardized computing constraints. The method ranking remains somewhat constant throughout the optimization. Only single curves, like LSTM-HC on DRD2 in the sample-constrained setting, show a significant increase in performance towards the end of the optimization. Similarly, this holds for AugmentedHC in the time-constrained setting.

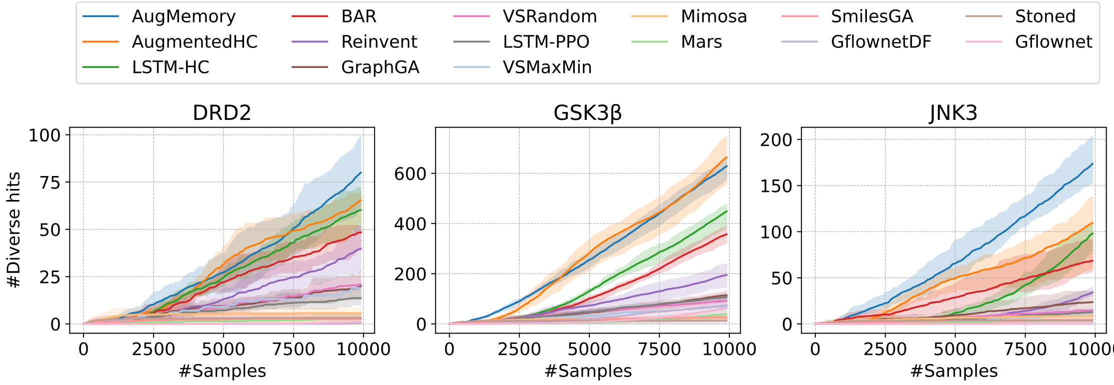

Figure S2: Number of diverse hits found by the tested methods over the number of scoring function evaluations.

S5.3 Cost to fixed performance

In Figure S4 we show the incurred compute costs to reach a fixed number of diverse hits. We chose this number such that most methods were able to reach them within the given compute constraints. This resulted in 20/100 diverse hits for the sample/time limit respectively.

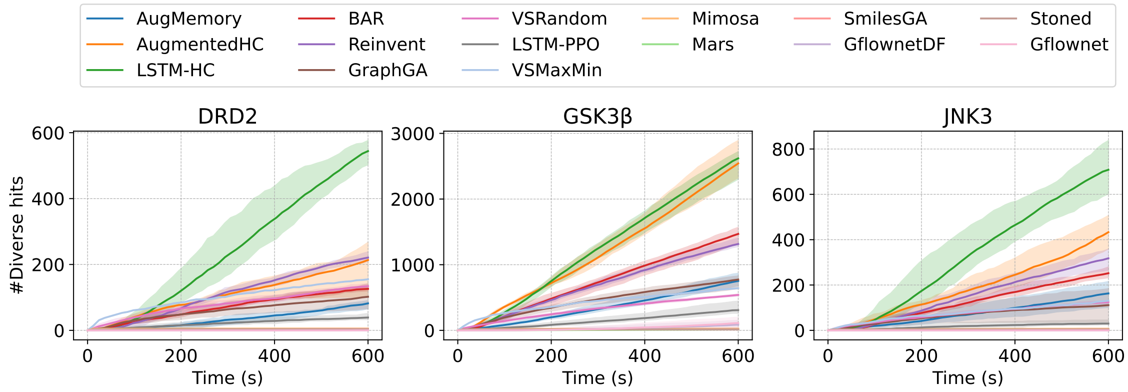

Figure S3: Number of diverse hits found by the tested methods over the elapsed time.

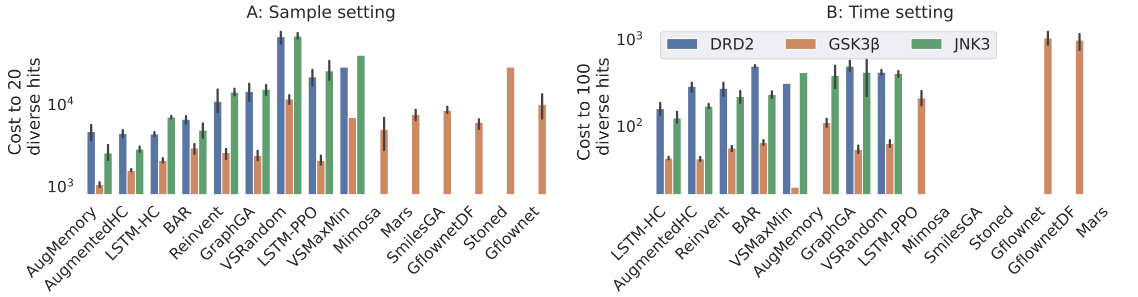

Figure S4: Compute cost to reach a fixed number of diverse hits. The cost is given in terms of the number of scoring function evaluations / time. Missing bars indicate that the method did not reach the threshold within the given compute constraints.

The difference in costs to reach the same number of diverse hits is very substantial with virtual screening methods requiring up to 10x the number of scoring function evaluations compared to the best performing methods in the sample-constrained setting. The differences are less pronounced in the time-constrained setting, where the factors are in the range from 3-5x.

The missing bars indicate that methods did not reach the threshold within the given compute constraints. These ommisions were necessary to keep the thresholds high enough for the results to be informative, for the rest of the methods. Running these remaining methods until they reach the threshold would have resulted in an excessive run time, as the limits are quite ambitious for the lower performing methods. The maximum values in this comparison serve as a lower bound for the missing values.

S5.4 Model ranking

To determine an overall ranking, we computed a combined ranking incorporating model performance in all six settings (2 compute limits $\times$ 3 tasks). In Figure S5 we show the average rank and the mean normalized performance for each algorithm. The mean normalized performance is computed as an average of an algorithm's performance divided by the best performance for the respective task and compute limit.

LSTM-HC demonstrates the highest mean normalized performance obtaining 84%, followed by AugmentedHC at 74% and AugMemory at 60%. This makes LSTM-HC the most versatile of the optimizers. However, it is crucial to ensure that the hyperparameters for LSTM-HC are appropriately adapted to the compute budget. Hyperparameters optimized for the sample-constrained setting will perform poorly in the time-constrained setting, and vice versa.

S5.5 Molecular property distributions

In this section, we show distributions of the molecular weight (MW), the water-octanol partition coefficient (logP), the fraction of *de novo* ECFP4 bits, the synthetic accessibility (SA), the quantitative estimate of drug-likeness (QED), and the length of the SMILES strings of the generated molecules. Figure S6 shows the distributions for the sample constrained setting, and Figure S7

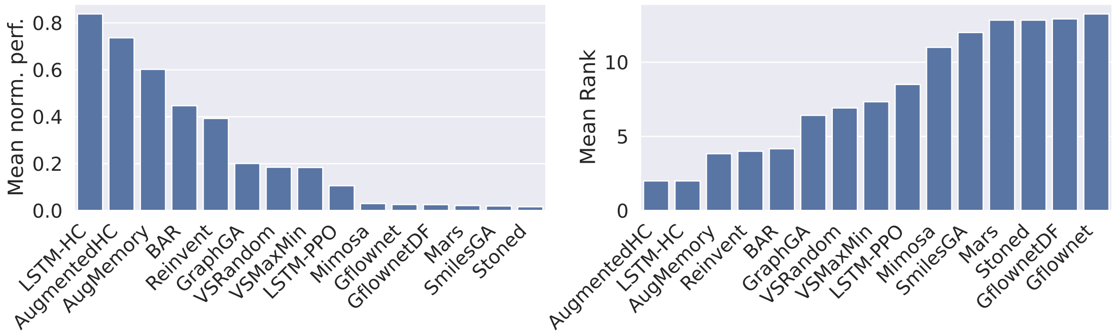

Figure S5: Model ranking in terms of mean normalized performance and average rank across all six tasks. LSTM-HC and AugmentedHC perform best and are most suitable as one-size-fits-all algorithms

shows the distributions for the time-constrained setting. For the properties used in the property filter, we also show the used thresholds.

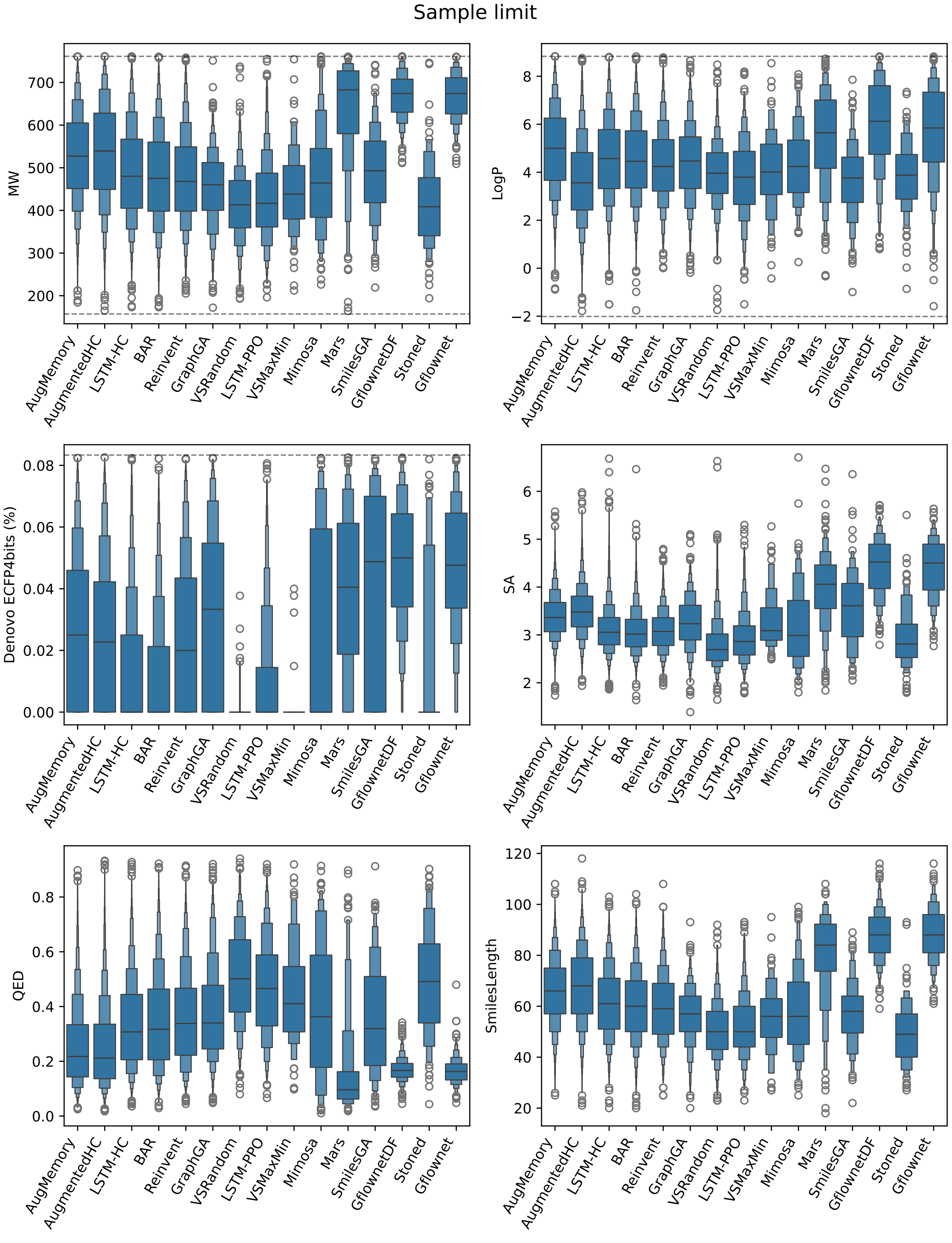

Figure S6: Distributions of the molecular properties of the generated molecules for the sample-constrained setting. The dashed lines indicate the thresholds used in the property filter. Results are aggregated over the three optimization tasks.

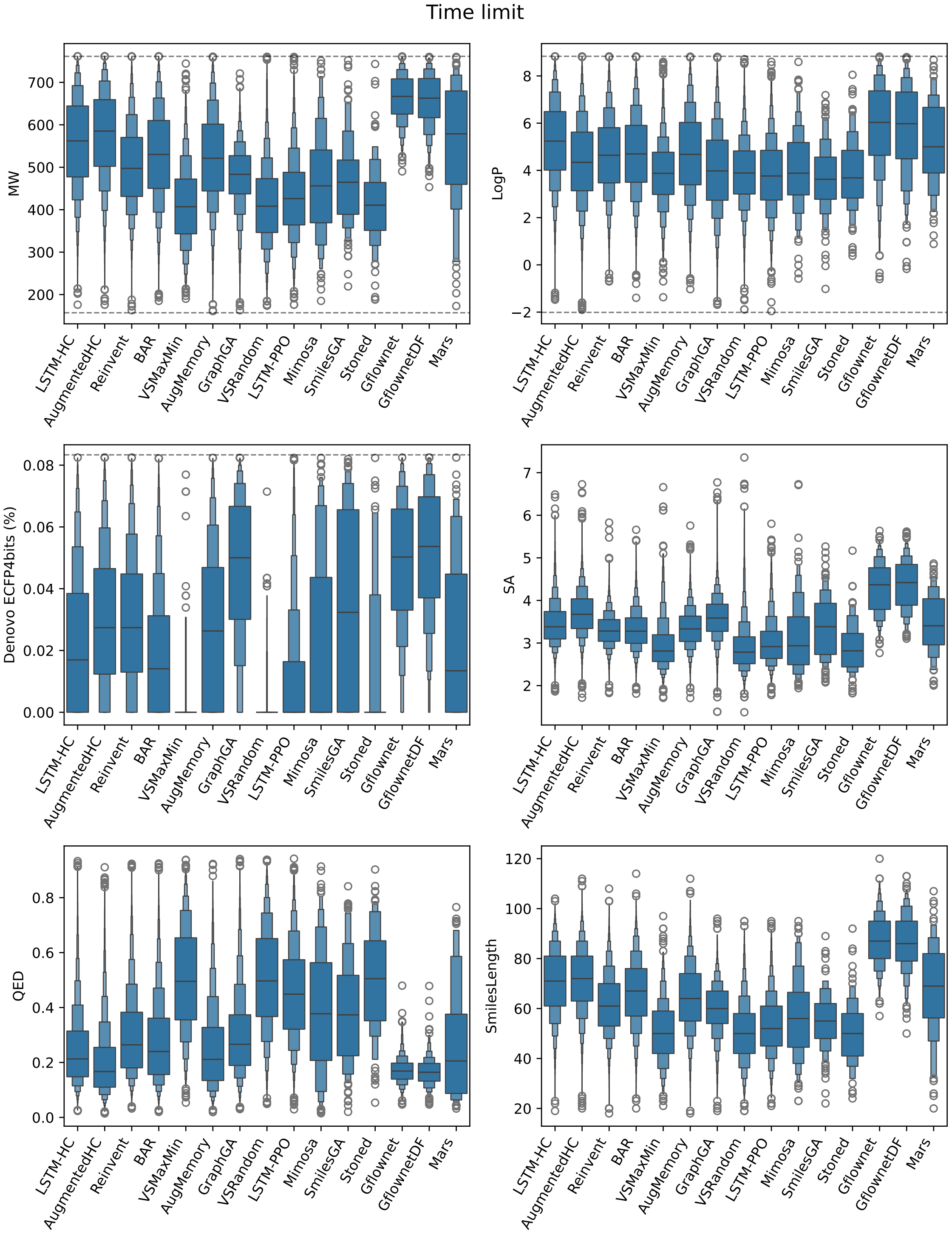

Figure S7: Distributions of the molecular properties of the generated molecules for the time-constrained setting. The dashed lines indicate the thresholds used in the property filter. Results are aggregated over the three optimization tasks.

References

[1] Jasial, S., Hu, Y., Vogt, M., Bajorath, J. "Activity-Relevant Similarity Values for Fingerprints and Implications for Similarity Searching". In: *F1000Res* 5 (2016), Chem Inf Sci–591. DOI: 10.12688/f1000research.8357.2.

[2] Sayle, R. *2D Similarity, Diversity and Clustering in RDKit*. 2019. URL: https://www.nextmovesoftware.com/talks/Sayle%5C%5C_2DSimilarityDiversityAndClusteringInRdkit%5C%5C_RDKITUGM%5C%5C_201909.pdf (visited on 11/17/2023).

[3] Landrum, G. *A New "Lessel and Briem like" Dataset*. URL: http://rdkit.blogspot.com/2019/10/a-new-lessel-and-briem-like-dataset.html (visited on 2023-10-26).

[4] Breiman, L. "Random Forests". In: *Mach. Learn.* 45.1 (2001), pp. 5–32. DOI: 10.1023/A:1010933404324.

[5] Rogers, D., Hahn, M. "Extended-Connectivity Fingerprints". In: *J. Chem. Inf. Model.* 50.5 (2010), pp. 742–754. DOI: 10.1021/ci100050t.

[6] Renz, P., Van Rompaey, D., Wegner, J. K., Hochreiter, S., Klambauer, G. "On Failure Modes in Molecule Generation and Optimization". In: *Drug Discov. Today: Technol.* Artificial Intelligence 32–33 (2019), pp. 55–63. DOI: 10.1016/j.ddtec.2020.09.003.

[7] Thomas, M., O'Boyle, N. M., Bender, A., De Graaf, C. "Re-Evaluating Sample Efficiency in de Novo Molecule Generation". In: (2022). DOI: 10.48550/arXiv.2212.01385.

[8] Brown, N., Fiscato, M., Segler, M. H., Vaucher, A. C. "GuacaMol: Benchmarking Models for de Novo Molecular Design". In: *J. Chem. Inf. Model.* 59.3 (2019), pp. 1096–1108. DOI: 10.1021/acs.jcim.8b00839.

[9] Blaschke, T., Engkvist, O., Bajorath, J., Chen, H. "Memory-Assisted Reinforcement Learning for Diverse Molecular de Novo Design". In: *J. Cheminformatics* 12.1 (2020), p. 68. DOI: 10.1186/s13321-020-00473-0.

[10] Weininger, D. "SMILES, a Chemical Language and Information System". In: *J. Chem. Inf. Comput. Sci.* 28.1 (1988), pp. 31–36. DOI: 10.1021/ci00057a005.

[11] Segler, M. H. S., Kogej, T., Tyrchan, C., Waller, M. P. "Generating Focused Molecule Libraries for Drug Discovery with Recurrent Neural Networks". In: *ACS Cent. Sci.* 4.1 (2018), pp. 120–131. DOI: 10.1021/acscentsci.7b00512.

[12] Olivecrona, M., Blaschke, T., Engkvist, O., Chen, H. "Molecular De-Novo Design through Deep Reinforcement Learning". In: *J. Cheminformatics* 9.1 (2017), p. 48. DOI: 10.1186/s13321-017-0235-x.

[13] Williams, R. J. "Simple Statistical Gradient-Following Algorithms for Connectionist Reinforcement Learning". In: *Mach Learn* 8.3-4 (1992), pp. 229–256. DOI: 10.1007/BF00992696.

[14] Thomas, M., O'Boyle, N. M., Bender, A., de Graaf, C. "Augmented Hill-Climb Increases Reinforcement Learning Efficiency for Language-Based de Novo Molecule Generation". In: *J. Cheminformatics* 14.1 (2022), p. 68. DOI: 10.1186/s13321-022-00646-z. (Visited on 09/04/2023).

[15] Guo, J., Schwaller, P. "Augmented Memory: Capitalizing on Experience Replay to Accelerate De Novo Molecular Design". In: (2023). DOI: 10.48550/arXiv.2305.16160. arXiv: 2305.16160.

[16] Atance, S. R., Diez, J. V., Engkvist, O., Olsson, S., Mercado, R. "De Novo Drug Design Using Reinforcement Learning with Graph-Based Deep Generative Models". In: *J. Chem. Inf. Model.* 62.20 (2022), pp. 4863–4872. DOI: 10.1021/acs.jcim.2c00838.

[17] Neil, D., Segler, M., Guasch, L., Ahmed, M., Plumbley, D., Sellwood, M., Brown, N. *Exploring Deep Recurrent Models with Reinforcement Learning for Molecule Design*. 2018. URL: https://openreview.net/forum?id=HkcTe-bR- (visited on 05/06/2019).

[18] Schulman, J., Wolski, F., Dhariwal, P., Radford, A., Klimov, O. "Proximal Policy Optimization Algorithms". In: (2017). DOI: 10.48550/arXiv.1707.06347.

[19] Jensen, J. H. "A Graph-Based Genetic Algorithm and Generative Model/Monte Carlo Tree Search for the Exploration of Chemical Space". In: *Chem. Sci.* 10.12 (2019), pp. 3567–3572. DOI: 10.1039/C8SC05372C.

[20] Gao, W., Fu, T., Sun, J., Coley, C. W. "Sample Efficiency Matters: A Benchmark for Practical Molecular Optimization". In: *NeurIPS*. 2022.

[21] Yoshikawa, N., Terayama, K., Honma, T., Oono, K., Tsuda, K. "Population-Based de Novo Molecule Generation, Using Grammatical Evolution". In: *Chem. Lett.* (2018). DOI: 10.1246/cl.180665.

[22] Nigam, A., Pollice, R., Krenn, M., Gomes, G. d. P., Aspuru-Guzik, A. "Beyond Generative Models: Superfast Traversal, Optimization, Novelty, Exploration and Discovery (STONED) Algorithm for Molecules Using SELFIES". In: *Chem. Sci.* 12.20 (2021), pp. 7079–7090. DOI: 10.1039/D1SC00231G.

[23] Krenn, M., Häse, F., Nigam, A., Friederich, P., Aspuru-Guzik, A. "Self-Referencing Embedded Strings (SELFIES): A 100% Robust Molecular String Representation". In: *Mach. Learn.: Sci. Technol.* (2020). DOI: 10.1088/2632-2153/aba947.

[24] Xie, Y., Shi, C., Zhou, H., Yang, Y., Zhang, W., Yu, Y., Li, L. "MARS: Markov Molecular Sampling for Multi-objective Drug Discovery". In: *ICLR*. 2021.

[25] Xie, Y., Xu, Z., Ma, J., Mei, Q. "How Much Space Has Been Explored? Measuring the Chemical Space Covered by Databases and Machine-Generated Molecules". In: *ICLR*. 2023.

[26] Fu, T., Xiao, C., Li, X., Glass, L. M., Sun, J. "MIMOSA: Multi-constraint Molecule Sampling for Molecule Optimization". In: *AAAI*. 2021.

[27] Bengio, E., Jain, M., Korablyov, M., Precup, D., Bengio, Y. "Flow Network Based Generative Models for Non-Iterative Diverse Candidate Generation". In: *NeurIPS*. 2021.

[28] Pereira, T., Abbasi, M., Ribeiro, B., Arrais, J. P. "Diversity Oriented Deep Reinforcement Learning for Targeted Molecule Generation". In: *J. Cheminformatics* 13.1 (2021), p. 21. DOI: 10.1186/s13321-021-00498-z.

[29] Bjerrum, E. J., Margreitter, C., Blaschke, T., de Castro, R. L.-R. "Faster and More Diverse de Novo Molecular Optimization with Double-Loop Reinforcement Learning Using Augmented SMILES". In: *J. Comput. Aided. Mol. Des.* 37.8 (2023), pp. 373–394. DOI: 10.1007/s10822-023-00512-6.

[30] Liu, X., Ye, K., van Vlijmen, H. W. T., IJzerman, A. P., van Westen, G. J. P. "An Exploration Strategy Improves the Diversity of de Novo Ligands Using Deep Reinforcement Learning: A Case for the Adenosine A2A Receptor". In: *J. Cheminformatics* 11.1 (2019), p. 35. DOI: 10.1186/s13321-019-0355-6.

[31] Chen, B., Wang, T., Li, C., Dai, H., Song, L. "Molecule Optimization by Explainable Evolution". In: *ICLR*. 2020.

[32] Rupakheti, C., Virshup, A., Yang, W., Beratan, D. N. "Strategy To Discover Diverse Optimal Molecules in the Small Molecule Universe". In: *J. Chem. Inf. Model.* 55.3 (2015), pp. 529–537. DOI: 10.1021/ci500749q.

[33] Graff, D. E., Shakhnovich, E. I., Coley, C. W. "Accelerating High-Throughput Virtual Screening through Molecular Pool-Based Active Learning". In: *Chem. Sci.* 12.22 (2021), pp. 7866–7881. DOI: 10.1039/D0SC06805E.

[34] Tripp, A., Simm, G. N. C., Hernández-Lobato, J. M. "A Fresh Look at De Novo Molecular Design Benchmarks". In: *NeurIPS-AI4Science*. 2021.
