# Supplementary material for: Diverse Hits in De Novo Molecule Design: Diversity-Based Comparison of Goal-Directed Generators
Source: J Chem Inf Model. 2024 Jul 19;64(15):5756–61. doi: 10.1021/acs.jcim.4c00519 (PMC11323242; doi:10.1021/acs.jcim.4c00519)
Supplement: Supplementary file 2 — ci4c00519_si_002.zip [file ci4c00519_si_002.zip › molecule_drawings/index.html]

# Generated diverse hits

## Time limit

| DRD2 | AugHC | AugMemory | BestAgentReminder | GA | Gflownet | GflownetDF | LSTM-HC | LSTM-PPO | Mars | Mimosa | Reinvent | SmilesGA | Stoned | VS\_MaxMin | VS\_Random |
| --- | --- | --- | --- | --- | --- | --- | --- | --- | --- | --- | --- | --- | --- | --- | --- |
| GSK3 | AugHC | AugMemory | BestAgentReminder | GA | Gflownet | GflownetDF | LSTM-HC | LSTM-PPO | Mars | Mimosa | Reinvent | SmilesGA | Stoned | VS\_MaxMin | VS\_Random |
| JNK3 | AugHC | AugMemory | BestAgentReminder | GA | Gflownet | GflownetDF | LSTM-HC | LSTM-PPO | Mars | Mimosa | Reinvent | SmilesGA | Stoned | VS\_MaxMin | VS\_Random |

## Sample limit

| DRD2 | AugHC | AugMemory | BestAgentReminder | GA | Gflownet | GflownetDF | LSTM-HC | LSTM-PPO | Mars | Mimosa | Reinvent | SmilesGA | Stoned | VS\_MaxMin | VS\_Random |
| --- | --- | --- | --- | --- | --- | --- | --- | --- | --- | --- | --- | --- | --- | --- | --- |
| GSK3 | AugHC | AugMemory | BestAgentReminder | GA | Gflownet | GflownetDF | LSTM-HC | LSTM-PPO | Mars | Mimosa | Reinvent | SmilesGA | Stoned | VS\_MaxMin | VS\_Random |
| JNK3 | AugHC | AugMemory | BestAgentReminder | GA | Gflownet | GflownetDF | LSTM-HC | LSTM-PPO | Mars | Mimosa | Reinvent | SmilesGA | Stoned | VS\_MaxMin | VS\_Random |
