# Supplementary material for: Diverse Hits in De Novo Molecule Design: Diversity-Based Comparison of Goal-Directed Generators
Source: J Chem Inf Model. 2024 Jul 19;64(15):5756–61. doi: 10.1021/acs.jcim.4c00519 (PMC11323242; doi:10.1021/acs.jcim.4c00519)
Supplement: Supplementary file 2 — ci4c00519_si_002.zip [file ci4c00519_si_002.zip › molecule_drawings/samples_JNK3/Gflownet.html]

# Sample limit, JNK3, Gflownet
